# How to calculate the dose of chemotherapy

**DOI:** 10.1038/sj.bjc.6600139

**Published:** 2002-04-22

**Authors:** H Gurney

**Affiliations:** Department of Medical Oncology and Palliative Care, Westmead Hospital, Westmead, 2145, Australia

**Keywords:** dose calculation, under-dose, body surface area

## Abstract

Body surface area-dosing does not account for the complex processes of cytotoxic drug elimination. This leads to an unpredictable variation in effect. Overdosing is easily recognised but it is possible that unrecognised underdosing is more common and may occur in 30% or more of patients receiving standard regimen. Those patients who are inadvertently underdosed are at risk of a significantly reduced anticancer effect. Using published data, it can be calculated that there is an almost 20% relative reduction in survival for women receiving adjuvant chemotherapy for breast cancer as a result of unrecognised underdosing. Similarly, the cure rate of cisplatin-based chemotherapy for advanced testicular cancer may be reduced by as much as 10%. The inaccuracy of body surface area-dosing is more than an inconvenience and it is important that methods for more accurate dose calculation are determined, based on the known drug elimination processes for cytotoxic chemotherapy. Twelve rules for dose calculation of chemotherapy are given that can be used as a guideline until better dose-calculation methods become available. Consideration should be given to using fixed dose guidelines independent of body surface area and based on drug elimination capability, both as a starting dose and for dose adjustment, which may have accuracy, safety and financial advantages.

*British Journal of Cancer* (2002) **86**, 1297–1302. DOI: 10.1038/sj/bjc/6600139 www.bjcancer.com

© 2002 Cancer Research UK

## 

Despite the recent advances in anticancer treatment and the promise of novel targeted therapies, it is likely that cytotoxic chemotherapy will continue to be used for the next few decades. It is now recognised that our current method of dose calculation for chemotherapy using body surface area (BSA) is inaccurate (Gurney, 1996, 1998; Ratain, 1998). This method does not account for the marked interpatient variation in drug handling that is known to exist for these drugs so that drug effects such as toxicity are also highly variable and therefore unpredictable. One consequence is unexpected underdosing which leads to reduced effectiveness of chemotherapy. However, until there is a better method, BSA-dosing will prevail since there has been over 40 years of experience with this method and ‘old habits die hard’. The following discussion will remind the clinician of the inaccuracies of this system and will suggest guidelines for dose calculation that encourages consideration of important parameters other than BSA alone.

To calculate dose accurately drug elimination needs to be understood. Typically there is a 4–10-fold variation in cytotoxic drug clearance between individuals due to differing activity of drug elimination processes related to genetic and environmental factors (Gurney, 1996). For example, the activity of cytochrome P450 (CYP) 3A4/5, the major oxidising enzymes for many cytotoxic drugs varies by as much as 50-fold (Wrighton *et al*, 1996). A common single-nucleotide polymorphism (SNP) or CYP3A5 has recently been identified and others are being searched for (Kuehl *et al*, 2001). In addition many drugs and disease states are known to inhibit or induce CYP activity further adding to this variation (George *et al*, 1996). Another example is the eight-fold variation in dihydropyrimidine dehydrogenase (DPD) activity, the enzyme that catabolises 5FU (Etienne *et al*, 1994). Less is known about the variation in other critical hepatic elimination processes such as active biliary excretion by multidrug resistance gene 1 (MDR1), multidrug resistance-associated protein 2 (MRP2) and the other ATP binding cassette (ABC) family of efflux pumps, although some polymorphisms have been identified (Tanabe *et al*, 2001). A number of SNPs have also recently been identified for the steroid and xenbiotic receptor (SXR), a common-pathway receptor which transcriptionally activates a number of the drug elimination genes such as CYP3A4, MRP2 and MDR1 (Zhang *et al*, 2001). Variation in renal function is more easily identified but none of these complex processes are accounted for when BSA alone is used to calculate drug dose.

## THE PROBLEM OF UNDERDOSING

It is clear that for most cancers there is a plateau in the dose–response curve for cytotoxic chemotherapy. Increasing the dose above a standard dose will increase toxicity, but does not improve anti-tumour effect (Gurney *et al*, 1993; Stadtmauer *et al*, 2000). High-dose chemotherapy is now largely reserved for acute leukaemia and aggressive lymphomas in relapse. However, the dose intensity studies from the last two decades have shown that anti cancer effect is substantially reduced if the dose of drug is intentionally decreased below the standard. What has not been recognised is that a significant proportion of patients may be *inadvertently* underdosed because of our inaccurate dose–calculation methods, which may cause a reduced cure or other effect.

**Figure 1:**
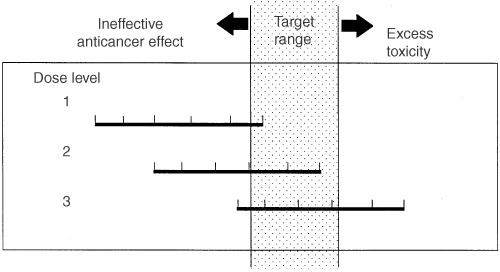
Hypothetical phase I study of a drug with linear pharmacokinetics. Horizontal bars represent interpatient variation in systemic exposure. Each vertical tick mark represents an individual patient on the study. Dose level 3 would be considered the MTD and dose level 2 would be recommended for phase II study despite the majority of patients on that dose level having a systemic exposure in the sub-therapeutic range.

The dose of a new drug is conventionally determined in a phase I study and then adjusted after more widespread use. The end point of this process is prevention of toxicity rather than identifying the dose for best anti-tumour effect. One consequence of this, coupled with the inaccuracy of BSA-dosing, is that significant underdosing becomes intrinsic to our system of dose determination. Figure 1

illustrates the scheme of a phase I study for a drug with linear pharmacokinetics. The horizontal lines represent the variation in systemic exposure at various dose levels. At dose level 3, those patients with lower drug elimination capability develop dose-limiting toxicity and subsequently that dose level is defined as the maximum tolerated dose. Dose level 2 is recommended for phase II studies since it causes tolerable toxicity in all patients. However, due to the variation in drug handling, a proportion of patients will be relatively underdosed since they are more capable of eliminating the drug. This means the wide distribution of systemic exposure is skewed towards the ineffective range when dose is calculated using BSA. Evidence for this effect can be found in a recent study by Gaemelin *et al* (1999). This group had defined the optimum 5FU plasma concentration with a regimen using 5FU in a dose of 1300 mg m^2^ infused over 8 h every week. For a group of 81 patients treated with dose calculated using BSA, 80% of patients were found to have an ineffective 5FU plasma concentration after the first dose.

What other evidence is available to indicate that underdosing occurs with the current dose calculation method? Here the problem is in defining and identifying underdosing. Can the lack of effect on normal tissue (i.e. toxicity) be used to identify a lack of effect in neoplastic tissue? For this to be tenable a toxicity-response relationship must be shown for cytotoxic chemotherapy. There is a wealth of information regarding dose–toxicity and dose–response relationships but very little information is available in the literature examining the relationship between toxicity and response (Gurney *et al*, 1993). Three studies from the 1970s and 80s purport a relationship between lack of myelosuppression and lack of anti-tumour effect in osteosarcoma and multiple myeloma (Cortes *et al*, 1974; McIntyre *et al*, 1978; Carpenter *et al*, 1982). However, a firm relationship cannot be claimed given the low patient numbers and the technique of analysis of these studies.

More recently, some studies have illustrated a toxicity-response relationship for breast cancer, testis cancer, ovarian cancer and lymphoma (Table 1

**Table 1:**
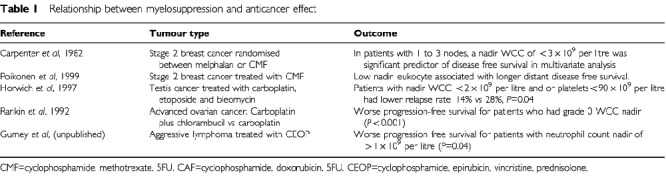
Relationship between myelosuppression and anticancer effect

) (Rankin *et al*, 1992; Horwich *et al*, 1997; Poikenen *et al*, 1999). A randomised MRC study of combination chemotherapy in advanced testicular cancer showed a significantly higher relapse rate in patients receiving carboplatin who failed to develop myelosuppression. There was a similar relationship shown for patients receiving cisplatin. Relapse rate for the cisplatin containing regimen was 11% for patients with a nadir white cell count (WCC) of over 2.0×10^9^ per litre compared with 4% for patients whose WCC fell below 2.0×10^9^ per litre after chemotherapy. Although this difference was not statistically significant, inadvertent underdosing may be an issue for cisplatin as well as carboplatin-containing regimen in the treatment of testicular cancer. These studies show a significantly worse anti-tumour effect for those patients who failed to develop myelosuppression after treatment compared to those who did. It is important that this relationship is examined more fully in other cancer types. This can be done by re-analysis of previous studies where nadir blood counts have been recorded in the majority of patients. A recent randomised study by the Australian Lymphoma and Leukaemia Group comparing high dose cyclophosphamide, epirubicin, vincristine and prednisolone (CEOP) with standard dose CEOP, showed that those patients who did not experience a nadir neutrophil count of <1.0×10^9^ per litre, had a statistically inferior progression free survival (Gurney *et al*, manuscript in preparation).

If lack of myelosuppression is accepted as an indication of underdosing, the frequency of this event can then be determined. Table 2

**Table 2:**
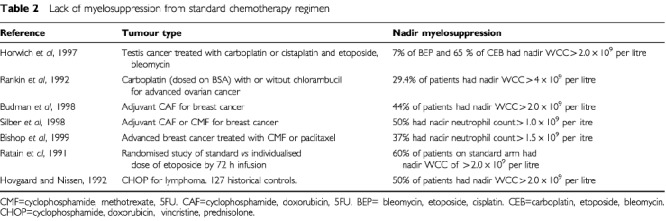
Lack of myelosuppression from standard chemotherapy regimen

is a selection of trials where the frequency and timing of nadir blood counts have been adequately recorded. A substantial percentage of patients (30 to 75%) receiving commonly used chemotherapy regimen have ‘inadequate’ myelosuppression and may be underdosed.

## THE SIGNIFICANCE OF UNDERDOSING

The possible significance of the underdosing is outlined in Table 3

**Table 3:**
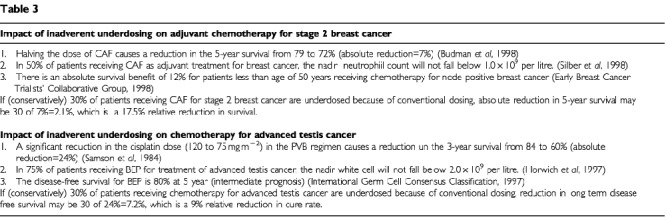

. Calculation of published data from studies using adjuvant cyclophosphamide, doxorubicin and 5FU (CAF) for node positive breast cancer show that BSA-based dosing may lead to almost a 20% relative reduction in survival in this setting (Budman *et al*, 1998; Early Breast Cancer Trialists' Collaborative Group, 1998; Silber *et al*, 1998). This impact is equivalent to the benefit from the use of adjuvant chemotherapy in node negative breast cancer, or the addition of paclitaxel to the CAF regimen in node positive breast cancer. Similarly, BSA-based dosing may reduce the cure rate of intermediate prognosis testis cancer by almost 10% compared to a dosing method that prevents underdosing (Samson *et al*, 1984; Horwich *et al*, 1997). Clearly, if these calculations are accurate, we can no longer tolerate the inaccuracies of BSA dosing as of minor consequence. It is important that more accurate calculation methods are developed. Another obvious bonus of an accurate dose calculation scheme is prevention of toxicity from overdosing.

## PREVENTION OF UNDERDOSING

One popular method of dose individualisation is to adjust subsequent doses of chemotherapy based on the level of myelosuppression eventually avoiding overdosing and underdosing – so called ‘toxicity-adjusting dosing’ (Gurney, 1996). A Swedish group has adopted this approach in the adjuvant treatment of breast cancer (Berch *et al*, 2000). Using the 5FU, epirubicin and cyclophosphamide (FEC) regimen, dose adjustments were made on each cycle to ensure a target level of myelosuppression. After a number of dose adjustments this method of individualising dose gave a three-fold interpatient range of cyclophosphamide dose (450 to 1800 mg m^2^) and a four-fold range for epirubicin (38 to 120 mg m^2^). This is more in keeping with the known interpatient variation in drug clearance for these drugs. However, it would be better to achieve an individualised dose variation from the first dose rather than the third, fourth or fifth dose. To achieve this, the dose calculation method must take into account the activity of the elimination processes for the drug(s) in question before the first treatment is given.

## FIXED DOSE?

Until better dose calculation methods are determined most clinicians will continue with the traditional method using BSA. However, clinicians should be mindful of the inaccuracies of this system and should not be duped by its pseudo-scientific use of formulas and slide rules. Doses should be rounded liberally. Fractional doses are irrelevant and unnecessary. Furthermore they are expensive and possibly unsafe. It is unreasonable to use a small portion of an extra vial of chemotherapy if the dose prescribe is inaccurate 40% of the time. Ask your pharmacist whether he/she can really draw up 215 mg of DTIC (instead of 200 or 220 mg), 85 mg of docetaxel (instead of 80 or 90 mg) or 63 mg of methotrexate. What is the additional cost of prescribing 305 mg of paclitaxel instead of 300 mg?

Consideration should be given to using a range of ‘fixed doses’ for a particular drug that could be used as the starting dose and for dose adjustments. Remember that drug elimination varies by at least four-fold between individuals. Can a clinically significant different pharmacodynamic effect be expected between 650 and 700 mg of 5FU? This probably holds true even for carboplatin where doses are determined as a function of glomerular filtration rate. There is still a margin of error in these calculations so dose rounding is also tenable in this situation. The alternative of using a fixed dose for chemotherapy has recently been suggested for cisplatin and irinotecan after investigators found no relationship between BSA and clearance for both of these drugs (de Jongh *et al*, 2001; Mathijssen *et al*, 2002).

## GUIDELINES

Guidelines for dose calculation are listed in Table 4

**Table 4:**
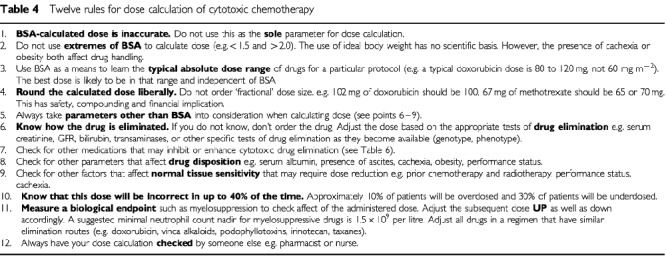
Twelve rules for dose calculation of cytotoxic chemotherapy

and an example in Table 5

**Table 5:**
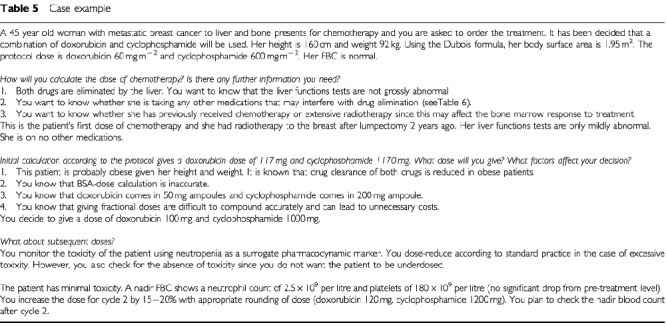
Case example

. These are not comprehensive and should be used in conjunction with clinical experience and good clinical practice. Some of them are subjective and based on opinion and derived from clinical practice while others are based on best evidence as reviewed in Gurney (1996). The guidelines allow a framework in which to work, in an area currently fraught with uncertainty. As other methods of dose calculation become available, they can be tested against these guidelines and adopted into clinical practice if found to be superior.

As yet there are no useful *in vivo* measures of drug elimination that can be used for dose calculation. Efforts have been aimed at predicting alteration in drug elimination in those with grossly abnormal liver or renal function with limited success. Carboplatin can be fairly accurately dosed by measuring the GFR. Guidelines exist for dose adjustment of other cytotoxic drugs that are predominantly renally excreted (Kintzel and Dorr, 1995). However, most cytotoxic drugs are largely hepatically eliminated. Attempts at using elevation of serum transaminases and alkaline phosphatase as a guide to dose adjustment have largely failed except perhaps for docetaxel (Alexandre *et al*, 2000).

Potential drug interactions is an extensive problem and warrants a separate review. Drug elimination can be enhanced by activation of the steroid xenobiotic receptor (SXR) and other nuclear receptors (Synold *et al*, 2001; Kast *et al*, 2002). SXR has multiple ligands including rifampicin, dexamethasone, cyproterone acetate, spirinolactone, St John's wort and others. SXR activation leads to upregulation of transcription of many elimination pathways including CYP3A4/5, 2B6, 2C8, MDR1, MRP2 and glutathione-s-transferase. Inhibition of CYP enzymes and MDR1 and probably other efflux pumps can occur with drugs such as cyclosporin, HMGCoA reductase inhibitors, verapamil, omeprazole and cimetidine. However, few clinically significant interactions have been documented or examined for cytotoxic chemotherapy. Anti-convulsant induction of CYP3A4 (phenytoin, phenobarbitone, carbemazepine) has been shown to affect the pharmacodynamics of paclitaxel, irinotecan and tenipisode and concomitant administration of anti-convulsants with chemotherapy has been associated with a worse disease-free survival in children with acute lymphoblastic leukaemia (Chang *et al*, 1998; Friedman *et al*, 1999; Relling *et al*, 2000). Table 6

**Table 6:**
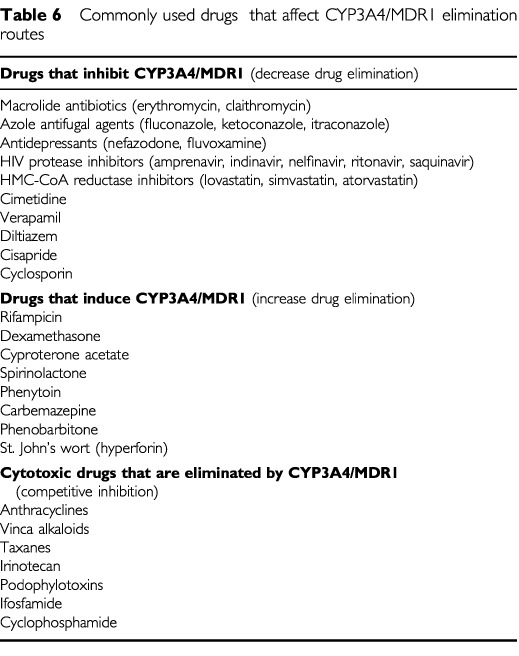
Commonly used drugs that affect CYP3A4/MDR1 elimination routes

lists commonly used drugs which may interfere with cytotoxic drug elimination, focusing on the CYP3A4/MDR1 axis since this is the major elimination route for most cytotoxic drugs and the site of most potential drug interactions.

## THE FUTURE

Studies are underway to define the drug handling genotype and phenotype *before* drug administration so an individualised dose can be given on the first cycle (Gurney *et al*, 1998, 2001; Kuehl *et al*, 2001; Tanabe *et al*, 2001; Schott *et al*, 2001; Zhang *et al*, 2001). Assessment of both hepatic metabolism and active biliary excretion is essential since these are the important elimination processes for the majority of cytotoxic drugs. Such *in vivo* tests of drug handling would have the advantage of being applicable to a range of cytotoxic and non-cytotoxic drugs, cleared by similar mechanisms.

One scenario is that the majority of patients who have ‘normal’ drug elimination receive a standard fixed dose of drug according to the regimen. Pretreatment *in vivo* tests of genotype or phenotype will identify the estimated 20 to 30% of patients who fall into the extremes of drug elimination capability. These patients will receive significantly lower or higher fixed doses. In other words, starting doses will be a range of fixed doses according to low, normal or high drug elimination. Fine-tuning of doses will be based on the presence or absence of toxicity or some other parameter that measures biological effect.

BSA-dosing can no longer be viewed as an inaccuracy causing minor inconvenience in treatment of cancer patients. We have the means to solve this problem and it is important that we do so swiftly. Identification of drug handling capability before treatment can allow the abandonment of BSA-dosing and avoid serious but often unrecognised underdosing.
